# Enzyme-modified non-oxidized LDL (ELDL) induces human coronary artery smooth muscle cell transformation to a migratory and osteoblast-like phenotype

**DOI:** 10.1038/s41598-018-30073-w

**Published:** 2018-08-10

**Authors:** Bijoy Chellan, Elizabeth Rojas, Chunling Zhang, Marion A. Hofmann Bowman

**Affiliations:** 1 Department of Medicine, University of Chicago, Chicago, IL USA; 2 University of California, Los Angeles, CA USA; 3 Center for Research Informatics, University of Chicago, Chicago, IL USA; 4 Department of Medicine, University of Michigan, Ann Arbor, MI USA

**Keywords:** Cell migration, Mechanisms of disease

## Abstract

Enzyme modified non-oxidative LDL (ELDL) is effectively taken up by vascular smooth muscle cells (SMC) and mediates transition into foam cells and produces phenotypic changes in SMC function. Our data show that incubation of human coronary artery SMC (HCASMC) with low concentration of ELDL (10 μg/ml) results in significantly enhanced foam cell formation compared to oxidized LDL (200 μg/ml; p < 0.01) or native LDL (200 μg/ml; p < 0.01). Bioinformatic network analysis identified activation of p38 MAPK, NFkB, ERK as top canonical pathways relevant for biological processes linked to cell migration and osteoblastic differentiation in ELDL-treated cells. Functional studies confirmed increased migration of HCASMC upon stimulation with ELDL (10 μg/ml) or Angiopoietin like protein 4, (ANGPTL4, 5 μg/ml), and gain in osteoblastic gene profile with significant increase in mRNA levels for DMP-1, ALPL, RUNX2, OPN/SPP1, osterix/SP7, BMP and reduction in mRNA for MGP and ENPP1. Enhanced calcification of HCASMC by ELDL was demonstrated by Alizarin Red staining. In summary, ELDL is highly potent in inducing foam cells in HCASMC and mediates a phenotypic switch with enhanced migration and osteoblastic gene profile. These results point to the potential of ELDL to induce migratory and osteoblastic effects in human smooth muscle cells with potential implications for migration and calcification of SMCs in human atherosclerosis.

## Introduction

Enzyme-modified non oxidized LDL (ELDL) and oxidized LDL (OxLDL) are two prominent post-translational modification of low density lipoproteins (LDL) and are well characterized in their ability to mediate atherosclerosis^1–4^. Both, macrophages and smooth muscle cells (SMC) take up cholesterol and thereby form foam cells; with recent studies showing that as many as 50% of foam cells in human and murine lesions originate from SMC^5,6^. ELDL and OxLDL are rapidly taken up by macrophages, however, the mechanism how lipids transforms SMCs into foam cells is less studied. We recently demonstrated that ELDL is more potent than native LDL, OxLDL or acetylated LDL in inducing foam cells in murine SMC, and implicated macropinocytosis rather than receptor-mediated cholesterol uptake as the main mode of ELDL uptake in murine SMCs^7^. ELDL induces SMC activation and contributes significantly to a phenotypic switch of smooth muscle cells with gain of function for IL-6 secretion, proliferation and migration^8^.

ELDL is a modification of LDL which occurs through the action of hydrolytic enzymes and it differs from OxLDL in that it lacks oxidized lipids^9^. ELDL has been detected in human calcific aortic valve disease^10^ and in atherosclerotic lesions^11–13^. The prevailing hypothesis is that ELDL is produced locally in the vessel wall from native LDL via enzymatic modifications stemming from cells native to the vessel wall together with infiltrating immune cells^14^. For our *in-vitro* experiments we generate ELDL as previously reported by digestion of LDL with trypsin and cholesteryl ester hydrolase, with trypsin cleaving the apo B protein, thereby facilitating access for cholesteryl ester hydrolase to the lipid core^7^. Importantly, cholesteryl ester hydrolase is present in human arterial plaques at concentrations high enough for direct detection by immunostaining^15,16^. Potential candidates for proteolytic enzymes that may modify LDL *in vivo*, similar to that by trypsin *in vitro*, include plasmin, chymases, matrix metalloproteinases (MMPs) and cathepsins; all are highly expressed in atherosclerotic plaques^1,17,18^, with studies showing that both, plasmin and MMPs, are sufficient to produce ELDL *in vitro*^1^.

Migrating medial SMC are characteristic feature in atherogenesis and by undergoing osteoblastic transformation critically contribute to vascular calcification^19^. Depending on local factors, SMCs may form a protective cap overlying the atherosclerotic plaque or if dysfunctional, could promote plaque erosion and rupture, leading to thrombosis and myocardial infarction^3,20,21^. Multiple factors contribute to SMC migration and calcification including growth factors, cytokines, and remodeling of extracellular matrix by proteases released from inflammatory cells present within the atherosclerotic milieu^19,22–24^. OxLDL has been shown to activate SMC migration and calcification^25,26^; however the role of ELDL in SMC calcification is not studied.

Taken together, our studies demonstrate that ELDL is sufficient to change expression levels of genes that regulate calcification and migration, and together with the invitro studies we demonstrate augmented calcification and migration upon stimulation with ELDL, suggesting that ELDL can produce classic changes in SMC function that are characteristic of atherosclerotic lesions.

## Results

### ELDL induces foam cells in cultured human coronary artery smooth muscle cells (HCASMC)

**Figure 1:**
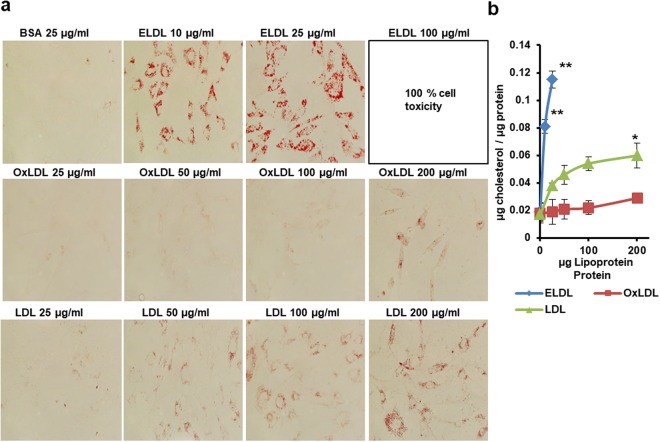
*Foam cell formation in human coronary artery smooth muscle cells*. (**a**) cells were incubated with either BSA, ELDL, OxLDL or Native LDL for 24 h at the indicated amounts (concentration by protein content) and lipid accumulation in SMC is visualized by Oil red O staining. (**b**) Quantitative analysis of cholesterol in SMC upon treatment for 24 h with indicated amounts of lipoprotein protein. Data are represented as mean ± SD. **p < 0.01 ELDL (for both 10 and 25 μg/mL) vs control BSA, *p < 0.05 for native LDL (200 μg/mL) vs control BSA.

ELDL was previously shown to be more potent than OxLDL in inducing foam cells in cultured macrophages^9^; and more recently, our laboratory demonstrated its ability to foam murine aortic SMCs at lower concentrations compared to OxLDL or native LDL^7^. However, such comparative studies in human SMC are lacking. We cultured human coronary artery smooth muscle cells (HCASMC) for 24 h with ELDL, OxLDL or native LDL in indicated concentration of lipoprotein (based on protein content) and the cellular lipid content was visualized by staining with Oil Red O after 24 hours as shown in Fig. 1a. As expected, ELDL was very effectively taken up by HCASMCs and induced foam cell formation at low concentration of 10 µg/ml. ELDL at 10 and 25 μg/mL lipoprotein-protein induced dose dependent accumulation of lipid, whereas cell death was observed at 100 μg/mL of ELDL, probably due to high cholesterol toxicity. However, substantially higher concentrations of OxLDL (200 μg/mL) were required for foam cell formation. The small amount of intracellular lipid accumulation induced by OxLDL at 200 μg/mL was further associated with significant cell death as evidenced by trypan blue staining (≈75%, data not shown). Unmodified native LDL induced foam cells formation at 100 and 200 μg/mL and no cytotoxicity was observed (data not shown). Taken together, total cell cholesterol content was significantly increased in HCASMC upon treatment with ELDL compared to OxLDL and native LDL when compared to same amount of lipoprotein based on protein measurement of LDL as shown upon quantification in Fig. 1b. Moreover, similar results were obtained upon quantification of lipoproteins based on cholesterol content after taking into account that ELDL has slightly more cholesterol per 10 µg protein concentration (Supplementary Figs 1 and 2). ELDL uptake and foaming of HCASMCs induced gene expression of some adipocyte markers (FABp4, perilipin), although many others genes for adipocytes or smooth muscle cell were similarly expressed in ELDL and BSA control cells (Supplementary Fig. 3).

### ELDL and oxidized LDL elicit distinct transcription networks in HCASMC

**Figure 2:**
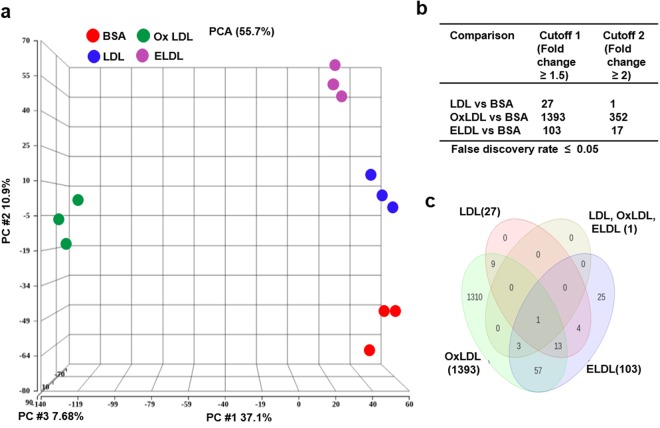
*Whole genome gene array profiling of HCASMC treated with LDL, OxLDL and ELDL*. (**a**) Principle component analysis (PCA) mapping for native LDL, OxLDL and ELDL treated HCASMC samples. (**b**) Number of differentially expressed genes (DEG) for native LDL, OxLDL and ELDL treated HCASMC. (**c**) Overlap map of DEG for native LDL, OxLDL and ELDL treated HCASMC versus BSA.

We next studied the effect of cholesterol treatment on gene expression in cultured HCASMC. HCASMC were stimulated with 50 μg/mL cholesterol (either as native LDL, oxidized LDL, or ELDL) and compared to BSA incubated control HCASMC. Whole genome gene expression was profiled using microarray chips (Illumina Bead Chip HT-12v4 array containing more than 47.000 probes). All samples passed basic quality control and the principle component analysis (PCA) showed a similar gene expression pattern for each of the three biological replicates of the four major treatment groups (Fig. 2a). Differentially expressed genes (DEGs) for each comparison were created for hierarchical clustering visualization. We analyzed data with cutoff criteria for gene expression ≥1.5 fold change and ≥2 fold change with a false discovery rate of <0.05. For the 1.5 fold cutoff criteria, 27 genes in cells treated with native LDL, 1393 genes for OxLDL, and 103 genes for ELDL treated cells were differentially expressed (Fig. 2b). The number of genes overlapping the four groups of differentially expressed genes is shown in Fig. 2c. Interestingly, of the 1393 genes differentially expressed in OxLDL treated cells, there were overlaps with only 74 genes in ELDL treated cells. Moreover, 1310 genes were only in the OxLDL list, and 25 genes only in the ELDL list. For example, the common subset of genes that belong to the intersect of DEGs detected in the LDL vs control (BSA) intersecting with OxLDL vs control BSA and ELDL vs control BSA is corresponding to only one gene. The criteria that was used to define the statistical significance of the detected DEG was that the FDR corrected p-value must be ≤0.05 and the absolute value of the fold change must be ≥1.5. From these only 3 belong to the intersection between OxLDL vs control and ELDL vs control, but these 3 don’t intersect with LDL vs. control as shown in Fig. 2c. Taken together, this suggests that gene expression is distinctly different between HCASMC stimulated with oxidized LDL compared to ELDL or native LDL. Hierarchical clustering for each of the three differentially expressed gene lists (OxLDL vs. control, native LDL vs. control and ELDL vs. control) are available in Supplementary Figs 4–6.

**Figure 3:**
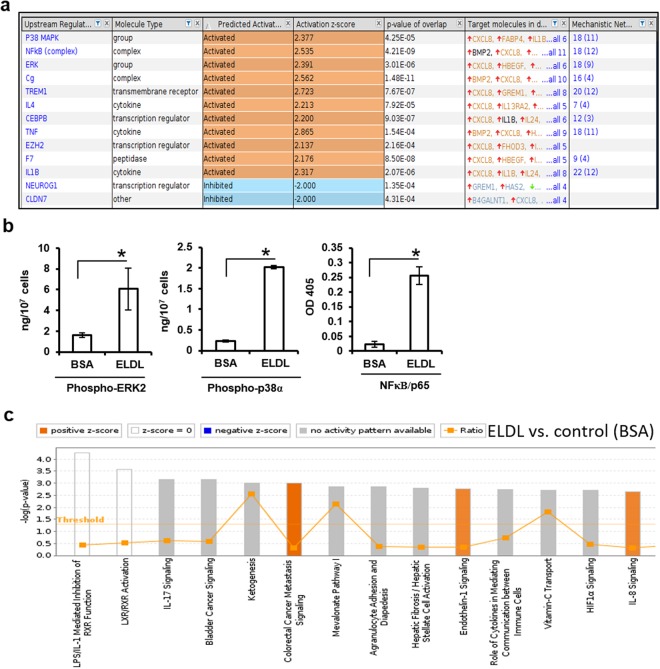
*Activation of p38 MAPK, NFkB and ERK in HCASMC stimulated with ELDL*. (**a**) Biological interpretations of differentially expressed genes in HCASMC treated with ELDL created by Ingenuity pathway enrichment analysis. (**b**) ELISA of top upstream regulators in ELDL treated HCASMC identified by Ingenuity pathways enrichment confirms activation of ERK2, p38α and p65 activity. Data are represented as mean ± SD, *p < 0.05 for ELDL vs control BSA. (**c**) Top canonical pathways in ELDL treated HCASMC predicted by Ingenuity pathway enrichment analysis.

We next explored functional annotation cluster using DAVID tools v6.8 and Ingenuity pathway enrichment analysis. The top three canonical pathways for ELDL vs. control are activation of p38 MAPK, NFkB, ERK and is shown in Fig. 3a. We next measured activation of phospho ERK2, phospho p38 and NFkB/p65 in whole cell lysis extracts prepared from HCASMC stimulated with ELDL (10 μg/ml) or BSA as control. As shown in Fig. 3b, all three signaling pathways were significantly activated in ELDL-stimulated cells.

Figure 3c presents the top canonical pathways affected by the list of Differentially Expressed Genes (DEGs) detected when comparing ELDL-treated smooth muscle cells versus control- treated smooth muscle cells. Each pathway is represented with its respective z-score, −log(p-value) and ratio. The calculated z-score indicates a pathway with genes exhibiting overall increased mRNA levels (orange bars), decreased mRNA levels (blue bars), no change (white bars), or no pattern available (grey bars). The calculated z-score is a statistical measure of the match between expected relationship direction and observed gene expression, if the z-score > 2 or <−2 it is considered *significant* by Ingenuity Pathway Analysis (IPA) tool. The ratio (orange dots connected by a line) indicates the ratio of genes from the dataset that map to the pathway, divided by the total number of genes that map to the same pathway. For ELDL-treated smooth muscle cells the top canonical pathways affected includes biological processes linked to cytokine activation (LPS/IL-1, IL17 signaling, IL-8 signaling), cell migration pathways (bladder cancer signaling, colorectal cancer signaling) and other (Fig. 3C). With the exception of IL-8 and IL-17, none of those pathways reached significant threshold in HCASMC treated with OxLDL or native LDL. As for oxLDL, the top canonical pathway was DNA damage checkpoint regulation (Supplementary Fig. 7), and NRF2-mediated oxidative stress response was the top canonical pathway for native LDL (Supplementary Fig. 8).

Taken together, this suggests that ELDL has unique properties in modulating gene expression in HCASMC. Activation of p38 MAPK, NFkB and ERK signaling was identified in the bioinformatics analysis as the most significantly upregulated upstream regulators and this was verified in cultured cells using ELISA assays for those signaling kinases. Furthermore, Supplementary Fig. 9 shows the network of cardiovascular system development and function for ELDL-treated HCASMC and demonstrates several nodes related to SMC-differentiation and calcification as shown by the canonical pathways of “Role of Osteoblast, Osteoclasts and Chondrocytes in Rheumatoid Arthritis”, “Role of Pattern Recognition Receptors in Recognition of Bacteria and Virus”, and “Atherosclerotic Signaling”.

### ELDL-mediated foam cell formation in cultured HCASMC up-regulates ANGPTL4 mRNA

**Figure 4:**
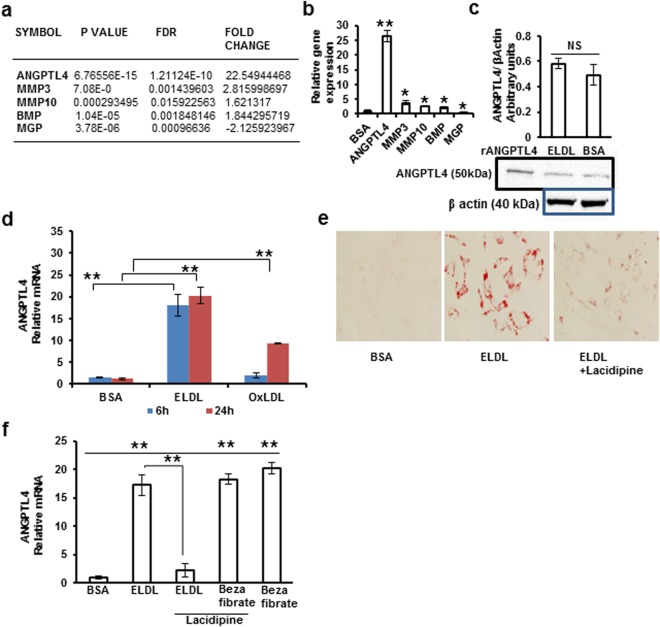
*ELDL upregulates ANGPTL4 mRNA in HCASMC more potently than OxLDL*. (**a**) fold change of gene expression of selected genes involved in cell migration and calcification from microchip gene array data of ELDL treated HCASMC. (**b**) qRT PCR analysis of mRNA of genes shown in a in HCASMC incubated with 10 μg/ml (by protein) of ELDL vs BSA for 24 h. Data are represented as mean ± SD, *p < 0.05 and **p < 0.01 vs control BSA. (**c**) Semi quantitative expression of ANGPTL4 protein in HCASMC and recombinant ANGPLT4 (25 ng) as control. (**d**) qRT PCR analysis of ANGPTL4 mRNA in HCASMC incubated with either 10 μg/ml (by protein) of ELDL or 25 μg/mL (by protein) of OxLDL vs control BSA at 6 h and 24 h. Data are represented as mean ± SD, **p < 0.01 for 6 h and 24 h ELDL vs control BSA and for 24 h OxLDL vs control BSA. (**e**) Foam cell formation assessed by Oil red O staining in HCASMC incubated for 24 hours with either 10 μg/mL ELDL ± 20 μM lacidipine (calcium channel blocker) or control BSA. (**f**) qRT PCR analysis of ANGPTL4 mRNA in HCASMC incubated for 24 hours with either 10 μg/mL ELDL ± lacidipine (20 μM) or control BSA or lacidipine (20 μM) ± bezafibrate (100 μM). Pharmacological agents were added 1 hour prior to adding ELDL. Data are represented as mean ± SD, **p < 0.01 for ELDL ± lacidipine vs control BSA and bezafibrate ± lacidipine vs control BSA.

Of the 103 genes differentially expressed in ELDL-treated cells, Angiopoietin like protein 4 (ANGPTL4) was one of the most up-regulated genes in the microarray data with a 22-fold increase (Fig. 4a). ANGPLT-4, MMP-3, MMP-10, bone morphogenic protein 2 (BMP2), and matrix gla protein (MGP) were validated by RT-PCR (Fig. 4b). Moreover, we found that ELDL induced a 20-fold upregulation of ANGPTL4 at 6 and 24 h, while OxLDL upregulated ANGPTL4 8-fold after 24 h, but not at the early time point of 6 h (Fig. 4d). This demonstrates that ELDL is very potent in inducing ANGPTL4 mRNA. However, there was no difference in ANGPLT4 protein expression in HCASMC stimulated with ELDL or BSA as shown by semi-quantitative immunoblotting (Fig. 4c).

ANGPTL4 mRNA is stimulated by fatty acids and may serve to protect cells against excess of fat uptake^27^. We therefore hypothesize that ELDL uptake potentially loads cells with lipids such as cholesterol and fatty acids and we tested whether lipid loading of HCASMC is required for up-regulation of ANGPTL4 mRNA. HCASMC were pretreated with lacidipine, a calcium channel inhibitor that was previously shown in our laboratory to abolish cellular uptake of ELDL in murine SMCs^7^. Similarly to murine SMCs, pretreatment of HCASMCs with lacidipine (20 µM) attenuates foam cell formation in HCASMC as shown in Fig. 4e. ANGPTL4 was originally identified as a PPAR α or γ induced protein^28,29^, and it was shown later that ANGPTL4 is also induced by PPAR β/δ^27,30^. We next examined the effect of lacidipine on mRNA expression for ANGPTL4 and found significant reduction of ANGPLT4 mRNA in HCASMC treated with lacidipine and ELDL, but no effect on ANGPLT4 mRNA induced by bezafibrate, a PPAR agonist (Fig. 4f). This suggests that ELDL uptake leading to foam cell formation is required for ELDL-mediated ANGPTL4 up regulation.

### ANGPTL4 up-regulation by ELDL is PPAR β/δ mediated

**Figure 5:**
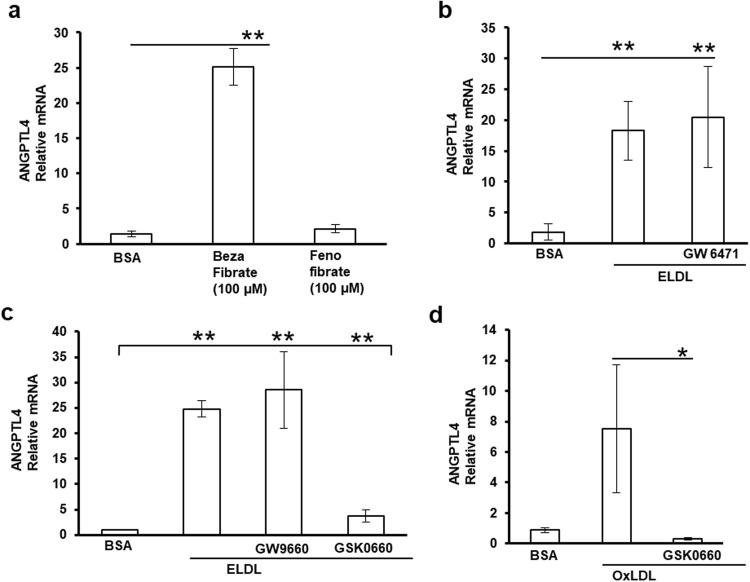
*ANGPTL4 upregulation by ELDL is PPAR β/δ mediated*. qRT PCR analysis of ANGPTL4 mRNA in HCASMC; all treatments were for 24 h and data are represented as mean ± SD; (**a**) HCASMC incubated with either control BSA or bezafibrate (100 μM) or fenofibrate (100 μM). **p < 0.01 for bezafibrate vs control BSA. (**b**) HCASMC incubated with either control BSA or 10 μg/mL ELDL ± GW6471 (25 μM, PPAR α inhibitor). **p < 0.01 for ELDL ± GW6471 vs control BSA. (**c**) HCASMC incubated with either control BSA or 10 μg/mL ELDL ± GW9660 (10 μM, PPAR γ inhibitor) or 10 μg/mL ELDL ± GSK0660 (10 μM, PPAR β/δ inhibitor). **p < 0.01 for ELDL ± GW9660 vs control BSA and ELDL vs ELDL + GSK 0660. (**d**) HCASMC incubated with either control BSA or 25 μg/mL OxLDL ± GSK 0660(10 μM). **p < 0.05 for OxLDL vs OxLDL + GSK 0660.

We further were interested in potential molecular mechanisms in ELDL- induced upregulation of ANGPTL4 in HCASMC. PPARs are regulators of lipid metabolism in cells, and unsaturated fatty acids are major ligands for all PPARs^31^ and we speculate that ELDL could potentially load HCASMC with such fatty acids. We therefore investigated whether ELDL-induced upregulation of ANGPTL4 in HCASMC is a PPAR dependent phenomenon. Synthetic PPAR agonist, fenofibrate is both a PPAR α and γ ligand with 10-fold selectivity for PPAR γ, while bezafibrate is a pan PPAR agonist^31^. When HCASMC were incubated with either bezafibrate or fenofibrate at 100 μM for 24 h, only bezafibrate upregulates ANGPTL4 mRNA (25 fold, Fig. 5a), indicating that ANGPTL4 mRNA upregulation in HCASMC is largely dependent on PPAR β/δ. To test this further, we employed several specific PPAR inhibitors and examined whether pretreatment with PPAR inhibitor attenuates ELDL-mediated increase in ANGPTL4 mRNA. Co-incubation of ELDL with GW6471, a specific PPAR α inhibitor did not change ANGPTL4 mRNA expression compared to that induced by ELDL alone (Fig. 5b). Further, we incubated ELDL with specific inhibitors of PPAR γ (GW9660) or PPAR β/δ (GSK 0660) and found that only GSK 0660 inhibited ELDL-induced upregulation of ANGPTL4 (Fig. 5c). This indicates that ELDL induced upregulation of ANGPTL4 is a PPAR β/δ dependent phenomenon. Moreover, PPAR β/δ inhibitor-GSK 0660 also inhibited upregulation of ANGPTL4 mRNA in cells co-incubated with OxLDL (Fig. 5d), supporting the hypothesis that loading of HCASMCs with fatty acids mediates the increase ANGPTL4 mRNA in HCASMCs.

### ELDL and rANGPTL4 induces migration in cultured human coronary artery smooth muscle cells

**Figure 6:**
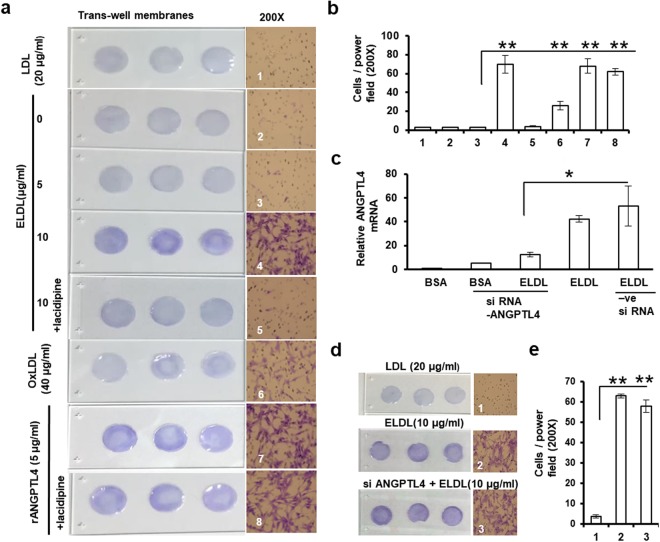
*ELDL and recombinant ANGPTL4 upregulates migration in cultured human coronary artery smooth muscle cells*. (**a**) HCASMC were treated as indicated for 24 h; lacidipine at 20 μM was added 1 h prior to adding ELDL or OxLDL. 25000 treated cells were seeded on the upper chamber of a transwell insert (pore size 8 μM, PET membrane) in a 12 well dish and cultured for 4 h while lower chamber had 20 ng/ml PDGF-BB as chemoattractant. Cells migrated to the lower side of the membrane were stained with crystal violet and assembled on glass slides for imaging. (**b**) Quantification of migrated cells by cell counting from 6 random high power fields (200X). Data are represented as mean ± SD. **p < 0.01 for 4, 6, 7, and 8 vs 3. (**c**) qPCR expression of ANGPTL4 mRNA in HCASMC treated with or without ELDL (10 µg/ml) for 24 hr with pretreatment either with siRNA for ANGPTL4 or vector control siRNA, ELDL was added 19 h after siRNA treatment. Data are represented as mean ± SD. *p < 0.01. (**d**) HCASMC were treated as indicated with ELDL with or without pretreatment with siRNA- ANGPTL4. (**e**) Quantification of migrated cells from panel d.

Growth factors, cytokines and extracellular matrix components promote migration of SMC in the atherosclerotic microenvironment within the vessel wall^22^. Modified LDLs are known to induce SMC migration and increased proliferation and migration of vascular SMC has been reported in response to OxLDL and ELDL^8,25,32^. HCASMC were incubated in dose-dependent manner with ELDL (0, 5 and 10 μg/ml) or OxLDL (40 μg/ml) for 24 h, after which cells (25000 cells in 100 μL media) were seeded on to the upper well of a transwell with an 8 μM porous membrane and cultured in media containing 20 ng/ml PDGF-BB in the lower well as chemoattractant. After 4 h in culture, cells on the upper side of the membrane were scrapped with a cotton swab, and the cells that migrated to the bottom side of the membrane were stained with crystal violet and subjected to quantification. As shown in Fig. 6a and quantified in b, ELDL upregulates migration of HCASMC, and is more potent than OxLDL. Since ELDL induces ANGPTL4 in HCASMC (Fig. 4) and ANGPTL4 promotes cell migration in experimental models of cancer^33,34^, we next tested whether recombinant ANGPTL4 protein stimulates migration of HCASMCs. We found that rANGPTL4 (5 μg/ml) significantly induces migration of HCASMC, and the effect was comparable to that by ELDL 10 μg/ml. Importantly, when HCASMC were co-incubated with ELDL and lacidipine (20 μM), migration of HCASMC was significantly reduced to basal levels indicating that ELDL uptake by the cells is necessary to induce migration in the HCASMC. In contrast, Lacidipine, did not influence rANGPTL4-induced migration of HCASMC (Fig. 6a,b).

Next, we tested whether ANGPTL4 is required for ELDL-induced HCASMC migration and utilized siRNA ANGPTL4 and control siRNA (RNAiMAX, LifeTechnologies). ANGPTL4-siRNA reduced ANGPTL4 mRNA by more than 70% in ELDL-treated cells, indicating that the siRNA was highly functional in inducing a net reduction of ANGPTL4 mRNA levels in ELDL treated HCASMC (Fig. 6c). However, siRNA mediated knockdown of ANGPTL4 mRNA in ELDL treated HCASMC did not prevent ELDL-induced HCASMC migration as shown in Fig. 6d,e. This indicates that ANGPLT4 is not required for ELDL-mediated migration of HCASMC. Moreover, siRNA mediated knockdown of ANGPTL4 in ELDL-treated cells had no effect on MMP3 and MMP10 mRNA expression in ELDL treated HCASMC (Supplementary Fig. 10). Taken together, our results indicate that both ELDL and rANGPTL4 upregulates migration in cultured HCASMC, and ANGPTL4 acts downstream of ELDL but is not required for ELDL-mediated cell migration.

We examined the effect of native LDL, ELDL and OxLDL on proliferation of HCASMC using two different assays, the MTT Proliferation assay (MTS/Formazan conversion) and BRDU incorporation. We found that native LDL and ELDL had no significant effect on proliferation of HCASMC and proliferation was similar to control cells treated with BSA. In contrast, OxLDL inhibited cell proliferation by approximately 20% (Supplementary Figs 11 and 12).

### ELDL upregulates phosphate induced calcification in cultured human coronary artery smooth muscle cells

**Figure 7:**
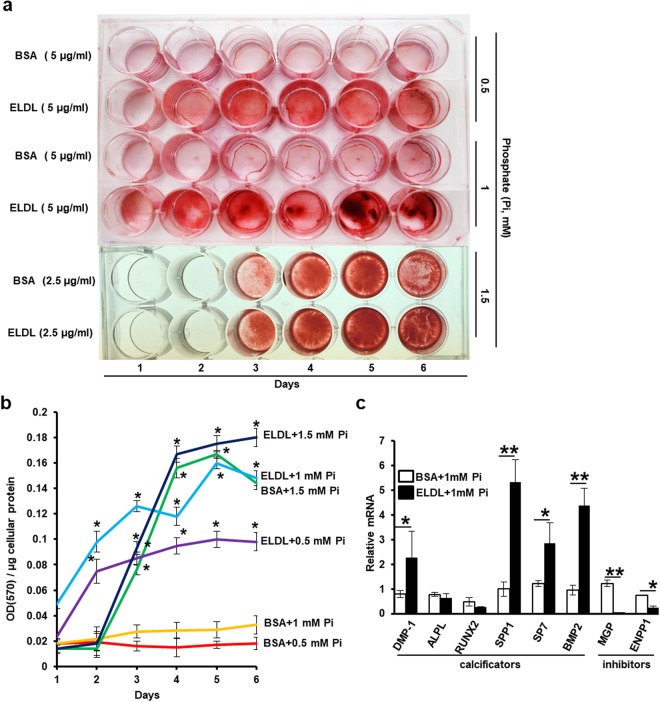
*ELDL upregulates phosphate induced calcification in cultured human coronary artery smooth muscle cells*. (**a**) Confluent monolayers of HCASMC cultured in phosphate containing pro-calcifying medium with either control BSA or ELDL as indicated. Calcium phosphate deposits were stained with Alizarin Red (200X images are shown in Supplementary Fig. 14). (**b**) Quantification of -Ca^2^ deposits from. Alizarin-Ca^2+^ complexes were extracted by the addition of 1 ml of 10% CPC in water. This extract was further diluted 1:10 before the optical density was read at 570 nm. Data are represented as mean ± SD, *p < 0.05 for ELDL (day 2–6) vs control BSA. (**c**) qRT PCR analysis of osteoblastic gene mRNA in HCASMC incubated for 24 hours with either 10 μg/mL ELDL or control BSA with 1 mM inorganic phosphate. Data are represented as mean ± SD, **p < 0.01 for ELDL vs control BSA in SPP1, BMP2 and MGP, *p < 0.05 for ELDL vs control BSA in DMP-1, SP7 and ENPP1.

Microarray gene expression profile of ELDL-induced HCASMC foam cells showed significant upregulation of mRNA for bone morphogenic protein 2 (BMP-2, ~ 2 fold), and downregulation of mRNA for matrix gla protein (MGP, >2 fold) as shown in Fig. 4a,b. Since BMP-2 and MGP are important proteins involved in vascular calcification, with MGP having regulatory roles for BMP-2 mediated vascular calcification^35–38^, and together with supporting data from the bioinformatics network analysis suggesting a role of ELDL for osteoblastic transformation as shown in Supplementary Fig. 9, we examined whether ELDL promotes calcification of HCASMC using Alizarin Red stain. ELDL alone (10 µg/ml) or in combination with organic phosphate beta Glycerophosphate (10–30 mM) in regular cell culture medium did not promote Alizarin Red staining when cultured up to 7 days (Supplementary Fig. 13), therefore inorganic phosphate at low concentrations from 0.5–1.5 mM was added to the cell culture medium to increase susceptibility to calcification. Consistent with previously reported findings^39^, we found no spontaneous calcification of HCASMC cultured in medium containing 0.5 and 1.0 mM phosphate. However, upon treatment with ELDL (5 μg/ml) to HCASMC cultured in medium containing 0.5–1.0 mM inorganic phosphate, there was significantly augmented mineralization of ELDL-treated cells as early as the 2^nd^ day of incubation (Fig. 7a). A higher magnification is provided in Supplementary Fig. 14. For quantification, the deposited Alizarin-Ca^2+^ complexes were extracted and the OD of the suitably diluted samples was read at 570 nm and normalized to total cellular protein. ELDL-treated cells showed significant more calcification compared to control (BSA) cells and this was evident on day 2–4 and plateaued by the 5^th^ and 7^th^ day of incubation (Fig. 7a,b). Since the effect was most noticeable at early time points, we tested the hypothesis that ELDL may alter gene expression of genes known to control osteogenic function in smooth muscle cells. mRNA was harvested from HCASMC cultured for 24 hours in the respective medium, at a time point without any morphological changes upon light microscopy nor increased Alizarin red staining. Moreover, there was no difference in mRNA quality and in the relative expression level for endogenous control genes HPRT and GAPDH (data not shown). Of the osteogenic genes known to be involved in vascular calcification, we selected genes either known to promote calcification (Dentin matrix acidic phosphoprotein 1, DMP-1; Alkaline phosphatase, ALPL; Runt-related transcription factor 2, RUNX2; Osteopontin, OPN/SPP1; osterix/SP7; BMP-2) or genes known as inhibitors of calcification (MGP, ectonucleotide pyrophosphatase/phosphodiesterase, ENPP-1) and measured their expression level by real time RT-PCR in HCASMC (Fig. 7c). ELDL in 1 mM phosphate medium upregulates pro-calcifying genes (DMP-1: 2 fold; SPP1: 5 fold; SP7: 2 fold; and BMP-2: 5 fold) and there was no difference ALPL and RUNX2. Interestingly, MGP mRNA and ENPP1 were strongly reduced (47-fold and 3 fold, respectively) by ELDL in 1 mM phosphate medium. Taken together our result indicates that ELDL significantly augments phosphate- induced calcification in cultured HCASMC by altering osteogenic gene expression.

## Discussion

The importance of ELDL and its atherogenic potential is now recognized as equally important along with other modified forms of LDL such as OxLDL^14^. While many studies have focused on macrophages as the main cell type in contributing to intimal foam cells, it is also known that smooth muscle cells can transform into foam cells *in vitro* and in human atherosclerotic lesions^5,40–42^. Here we show that human coronary artery smooth muscle cells avidly take up ELDL, and very low amounts of ELDL were sufficient to promote foam cell formation. To our knowledge, this is the first report for quantitative comparison of ELDL with other modified LDLs in inducing foam cells in HCASMC. Normal and atherosclerotic intima has been shown to contain 2 to 4 times higher content of native LDL than that is in circulation^43^. Since plasma LDL concentration is ≈1 mg/mL, intimal fluid may contain 2 mg/ml of native LDL. In our invitro experiments, foam cell formation by native LDL at 2 mg/mL was similar in intensity to that by 200 μg/mL native LDL (data not shown), although significantly less in comparison to ELDL at 10 μg/mL. Although ELDL has been detected previously in atherosclerotic lesions in the vasculature and in calcific aortic valve disease *in vivo*^10–13^, the concentration of ELDL in circulation is not known. Nevertheless, our invitro data suggests that ELDL is more potent than OxLDL or native LDL in inducing foam cells in cultured HCASMC.

*In vivo*, ELDL represents one of the many forms of modified LDLs that may form foam cells in macrophages and smooth muscles cells, and phenotypic modulations brought about by individual modified LDLs overlap significantly in an atherosclerotic milieu, making it practically impossible to differentiate between each other. By studying invitro-generated modifications of LDL we were able to demonstrate marked differences in the transcriptome of human SMCs upon uptake of ELDL, OxLDL and native LDL, indicating that specific modifications of LDL in the atherosclerotic plaques may determine the biology and functional consequences in vasculature.

We showed that MAPKp38, ERK and NFkB activation were the top three canonical pathways in an unbiased Ingenuity pathway enriched network analysis of ELDL-treated HCASMC, and these findings were confirmed by ELISA. This is in agreement with a recent study demonstrating co-localization of immunohistochemical staining for ELDL and p38MAK/MAPK14 in human carotid endarterectomy tissue from atherosclerosis with high grade stenosis^44^. Among the wide-ranging downstream activities of phosphorylated p38MAPK, cytokine production is a component related to atherosclerosis and osteoblast differentiation^45^. Indeed, biological network analysis indicates a potential role of ELDL in altering gene expression linked to calcification by (i) inhibition of calcification inhibitors, and by (ii) activation of osteogenic genes. To test whether ELDL promotes vascular calcification, we cultured HCASMC in phosphate-containing medium and found that addition of ELDL highly reduces gene expression of matrix gla protein and ENPP-1, together with up-regulation of other genes promoting calcification. Matrix gla protein is known as a major inhibitor of calcification^37,38^. ENPP1 is an enzyme that converts extracellular ATP to adenosine and thereby generates pyrophosphate, an important inhibitor of hydroxyapatite formation and calcification. Recessive loss of function mutation in ENPP1 leads to loss of enzyme activity, and to infantile arterial calcification^46,47^. The direct molecular mechanisms by which ELDL accelerates calcification of SMCs is not clear, but at least in part, it is linked to acquiring an osteoblastic gene profile in SMCs. Other lipoproteins including lipoprotein (a) and OxLDL have recently been shown to activate innate immune responses in interstitial valve cells leading to a gain of pro-calcific phenotypes in calcific aortic valve disease^48–50^.

Another important finding of our study is up-regulation of ANGPTL4 mRNA in HCASMC upon treatment with ELDL as demonstrated in the Illumina bead chip assay (22-fold increase) and confirmed by RT PCR. ANGPTL4 was recently shown to be expressed in murine coronary SMCs and cardiomyocytes in mice challenged with oral fatty acid bolus^30^, supporting the notion that our approach using *in-vitro* generated ELDL on cultured HCASMC sufficiently models *in vivo* processes. ANGPTL4 is a strong inhibitor of lipoprotein lipase and thought to prevent lipid toxicity to fat-loaded cells^27,30,51^. Therefore, up regulation of ANGPTL4 mRNA in HCASMC in response to ELDL is possibly as a physiological response to prevent lipid toxicity. Other roles of ANGPLT4 include regulation of hematopoiesis and cell migration in keratinocytes and fibroblasts^52,53^. However, while our data show induction of ANGPLT-4 mRNA in response to ELDL, we did not find an increase in ANGPLT4 protein level. Moreover, knockdown of ANGPLT-4 mRNA with siRNA did not prevent ELDL-mediated migration, suggesting that ANGPTL4 is not required for migration of HCASMC. ANGPTL4 has been studied in murine atherosclerosis. One study reported on decreased atherosclerotic lesion size in ApoE3-Leiden mice with transgenic overexpression of ANGPTL 4^30,54^, while Aryal *et al*. showed decreased atherosclerosis in LDLR null mice with global knock out of ANGPTL4 and accelerated atherosclerosis in LDLR null mice lacking ANGPTL4 only in the hematopoietic cells^55^. This suggests that ANGPTL4 has tissue specific effects with regards to atherosclerosis and our finding of enhanced migration of HCASMC by rANGPTL4 supports a pleotropic role of ANGPTL4 in regulation of non-metabolic processes.

Furthermore, coding variations in the ANGPTL4 gene has been studied in human populations, and for example a loss-of-function allele E40K in ANGPTL4 was associated with protection for myocardial infarction in the CARDIoGRAM exome consortium^56^, although associated with increased risk in another study^57^.

Lastly, we demonstrate that lacidipine attenuates uptake of ELDL in HCASMC. Lacidipine is a calcium channel blocker with antioxidative properties and we showed previously that it inhibits macropinocytosis of ELDL in a calcium and oxidative stress-dependent manner in murine SMCs^7,58^. We speculate that blocking ELDL-mediated foam cell formation of HCASMCs could be a potential therapeutic target for atherosclerosis. This hypothesis is supported by findings from a clinical trial, in which lacidipine (compared to atenolol) was shown to decrease carotid atherosclerosis in the European Lacidipine Study on Atherosclerosis with a greater efficiency on carotid intima–media thickness and number of plaques per patient despite a smaller reduction in ambulatory blood pressure, suggesting that the anti-atherogenic action of lacidipine is independent of its anti-hypertensive action^59^. A better understanding of these processes may lead to potential therapeutic options to inhibit ELDL uptake and smooth muscle foam cell formation.

In summary, we demonstrate that stimulation of HCASMC with ELDL promotes a “pro-atherosclerotic” phenotype by transforming smooth muscle cells into foam cells, associated with enhanced migration and gaining of osteoblastic gene expression. We speculate that this process could play a role *in vivo* since ELDL has been identified in atherosclerotic vasculature. Our data support the hypothesis that uptake of ELDL by SMCs could potentially lead to translocation of SMCs with altered phenotype, and by gaining of an osteoblastic gene profile may promote development of micro-calcification foci in SMC-rich areas of atherosclerotic plaques.

## Materials and Methods

### Lipoprotein Isolation and Modifications

LDL, ox LDL and ELDL was prepared as described previously by us^7^. Briefly, human plasma LDL (density 1.02–1.063 g/ml) was isolated by preparative ultracentrifugation under sterile conditions. To generate OxLDL, human LDL (5 mg) in 1 ml PBS containing CuSO4 (10 μM) was incubated for 48 h at 37 °C. The oxidized LDL was then dialyzed against PBS extensively, filter sterilized and stored at 4 °C. Protein content was measured by Bradford method. To generate Enzyme modified LDL (ELDL) 1 mg LDL was digested with 2 μg trypsin (Sigma) and 12 μg cholesterol esterase (Sigma) and incubated at 37 °C for 16 h, followed by adding another 2 μg trypsin and 20 μg cholesterol esterase per mg LDL cholesterol and incubation for 48 h at 37 °C. At the end of the incubation, the ELDL preparation looked cloudy and was extensively dialyzed (molecular weight cut off = 100 KD) against PBS. ELDL was filter sterilized and stored at 4 °C. Protein content was measured by Bradford method. ELDL was tested for trypsin content using trypsin activity assay kit (Abcam) and found to be similar to original LDL. Moreover, LDL and modified LDL’s were tested for endotoxin using the PYROGENT gel clot LAL assay (Lonza) and only negative samples were used for experiments.

### Human Coronary Artery Smooth Muscle cells (HCASMC)

HCASMC were purchased from Life technologies and cultured according to manufactures recommendation in Medium 231 supplemented with smooth muscle growth supplement (GIBCO). As per manufacturer information, cells were isolated by the enzyme digestion method as previously described by Ray *et al*.^60^. Expression of αSMA, a specific adult SMC marker, and absence of the specific endothelial cell protein CD31 was confirmed in HCASMC by immunoblotting using anti human SMA IgG (abcam) and anti human CD31 IgG (RD systems). As control cell line we used Human Aortic Endothelial cells, which abundantly express CD31 and was a kind gift by Dr.Yun Fang, Section of pulmonary and critical care, Department of Medicine, University of Chicago (Supplementary Fig. 15). Three different batches of HCASMC (from Life technologies) were propagated and passage 2–7 were used for experiments. With the exception for the microarray experiment, which was performed on HCASMC from only one donor, all other experiments utilized three different batches of HCASMC and each cell line was tested in triplicates.

### *In vitro* foam cell assay using Oil red O

Human coronary artery smooth muscle cells (Life Technologies) were cultured on 18 mm cover glass slips in 12-well dishes in media M231 supplemented with SMGS (SMC growth supplements, Life technologies) and 1% antibiotic-antimycotic (Gibco). BSA (control), native LDL, copper oxidized LDL or ELDL was added to cells in indicated dosages and incubated for 24 h at 37 °C. Where indicated, calcium channel inhibitor-lacidipine (Sigma) was added to the cells 1 h before the addition of ELDL for the entire incubation period of ELDL. Foam cell formation was assessed visually by Oil Red O staining intensity and quantified as a function of total cholesterol/protein in the cells.

### Oil Red O staining

0.35 g of Oil Red O (Sigma) was dissolved in 100 ml of isopropanol and stirred overnight at room temperature. Prior to use, the solution was filtered followed by dilution with water (volume 6:4) and filtered again immediately before applying to cells. Adherent cells were rinsed with PBS and fixed in 10% formalin at room temperature for 1 hour followed by rinsing with water. The cells were then incubated in 60% isopropanol for 5 minutes at room temperature and drained off. After complete drying of the cells, staining with Oil Red O for 10 minutes at room temperature was performed. The Oil red O was then removed and the cells were immediately rinsed with water 4 times. Cells were cover-slipped with mounting medium and the images were acquired with a light microscope at 600 X magnification.

### Whole genome gene expression and bioinformatics

HCASMC were cultured and stimulated with 50 μg/ml cholesterol (native LDL, ELDL, OxLDL) or bovine serum albumin for 24 hours. mRNA was harvested and 12 samples (three biological triplicates from 4 different treatment groups) were submitted to the Genomics Facility at the University of Chicago. Whole genome gene expression was profiled by microarray chips using the Illumina BreadChip HT-12v4 array. Bioinformatics analysis was performed at the Bioinformatics Core, Center for Research Informatics. Briefly, raw microarray data were processes by Illumina Genome Studio v2011.1 and Partek Genomics suite v6.6. Average signal intensities were background-subtracted, log2 transformed, and quantile normalized. A total 47,323 processed intensities and associated detection *p-values* at the probe level were obtained. The processed intensities at probe level were further filtered by detection p-value ≤ 0.01 in at least 3 samples. After filtering, 17,903 processed average signal intensities at probe level were used for the detection of differentially expressed genes (DEGs). Statistically significance for the DEGs was defined by the cut off of absolute value of fold change >1.5, and false discovery rate corrected p-value < 0.05. Detected differentially expressed genes and were further used for function annotation clustering analysis using DAVID tools v6.8 Beta and Ingenuity Pathway Analysis (IPA)”.

### Western Blots

Protein expression was semi-quantified by western blotting in whole cell lysis extracts from HCASMC using anti human ANGPTL4 antibody (abcam, ab115798), anti human CD31 IgG(RD systems), and anti human aSMA IgG (abcam).

### ELISA

phosphorylated ERK2, p38α and NFκB/p65 activity in HCASMC treated with ELDL or control were measured in whole cell lysis extracts using ELISA kits as per manufacturer’s instructions (ERK2 and p38α kits from RD sytems, NFκB/p65 kit from Novus biologicals). Cells were treated with 10 µg/ml ELDL for 4 hrs, BSA incubated cells were control.

### Quantitative real-time RT-PCR

Cells were preincubated with pharmacological agents such as Bezafibrate (100 µM), Fenofibrate (100 µM), GW 6471 (25 µM), GW 9660 (10 µM) and GSK 0660 (10 µM) (all Sigma) prior to adding modified LDLs as indicated. RNA was isolated from cells using TRIzol reagent (Invitrogen). First-strand cDNA was generated from 3 µg RNA using Superscript III (Invitrogen) and random primers (Invitrogen). Subsequently, the cDNA was diluted 1:10 and 2 µl cDNA was subjected to quantitative real time PCR using SYBR Green with a IQ5 cycler (BioRAD) with specific primers. All PCR amplifications were carried out in triplicates. The primers sequences are listed in Table 1. The ΔC_T_ value was used to describe the difference between the test and control cells normalized to the housekeeping gene HPRT. Relative mRNA expression was estimated as 2exp (ΔC_T_ target gene- ΔC_T_ housekeeping gene). There was no significant difference in absolute C_T_ values for the amplification of HPRT among the different experimental groups (C_T_ ranging from 19.59–20.52), indicating that RNA quality and abundance of this house keeping gene was not affected by the experimental design. si RNA mediated knockdown of ANGPTL4 was achieved using ANGPTL4 silencer RNA and control siRNA (Life Technologies). siRNA (25 pmole/2 ml/6well) was complexed with RNAiMAX(Life technologies) and added to cells as per manufacturer’s instruction. The end point ANGPTL4 mRNA expression was measured by qPCR.

**Table 1.** Primer sequences used for qRT-PCR.

Gene	Forward sequence	Reverse sequence
1. Human RUNX2	GCTTCATTCGCCTCACAAAC	GTAGTGACCTGCGGAGATTAAC
2. Human BMP-2	CCCACTTGGAGGAGAAACAA	CTAGCAATGGCCTTATCTGTGA
3. Human DMP1	GACCCACAAAGCTACAGAGTTA	CCTTCTCAGTGTTCCCAGATAG
4. Human SP7	GCCACACCAACACACTTTCTA	CTGAGGGACAGCAGGAAATAAG
5. Human ENPP1	CTTGCATTGAATCCCTCAGAAAG	AGGTCCATAGCCAACAAAGAG
6. Human MGP	CAGCAGAGATGGAGAGCTAAAG	GTCATCACAGGCTTCCCTATT
7. Human SPP1	CCGAGGTGATAGTGTGGTTTATG	CTTTCCATGTGTGAGGTGATGT
8. Human ALPL	GGAGTATGAGAGTGACGAGAAAG	GAAGTGGGAGTGCTTGTATCT
9. Human ANGPTL4	AGAAGACCACGACTGGAGAA	CGCCTCTAGAGTCTGAGCATA
10. Human HPRT	CCTGGCGTCGTGATTAGTGATGAT	AGCAAGACGTTCAGTCCTGTCCAT

### *In vitro* cell migration analysis

Human coronary artery smooth muscle cells (HCASMC) were cultured regularly in M231 media supplemented with SMGS. In invitro- cell migration analysis was performed by a modified method from that originally explained by Law *et al*. for cell migration towards a PDGF gradient^61^. Briefly, 50 ul of 1% complete growth media containing 20 ng/ml recombinant human PDGF-BB (BioLegend) was taken in a tissue culture well (12 well, Falcon) and a 8 µM transwell insert (Corning, transparent PET) was gently placed on to it. 25000 cells in 100 µl of 1% complete growth media were then placed in the transwell and cultured for 4 hrs. The cells were then gently scrapped off from the upper chamber/side of the transwell membrane using Q-tips (cotton swabs) and the transwell (with cells on the lower side of membrane) were fixed overnight in 10% formalin. Fixed transwells/cells were gently rinsed twice in distilled water and incubated for 15 minutes in 0.5% crystal violet in 25% methanol solution. Crystal violet was dissolved in methanol (100%) and then diluted with water to a final 25% methanol and solution was stirred for 15 minutes and filtered before adding to cells. Cells were washed several times gently using tap water until membranes are clear of background. Transwells were dried completely and the membranes were cut out from the transwell with a blade and assembled on a glass slide for microscopy.

### *In vitro* calcification assay

Human coronary artery smooth muscle cell (HCASMC) were cultured in calcification medium according to procedure modified from Shioi *et al*.^62^. Cells between passages 3 and 7 were used from three different batches of HCASMC (Life Technologies). Cells were grown to confluency in 12 well dishes using human SMC culture media (medium M231 supplemented with SMGS, both from Life technologies). The cells were then washed with PBS, and incubated overnight with Advanced MEM (Life technologies) supplemented with 0.2% of FBS, 1X antibiotic antimycotic (Anti Anti-Life technologies, Gibco). The next day, the medium was replaced with pro-calcifying medium (Advanced MEM with 0.2% of FBS, 1X antibiotic- antimycotic containing 0.5–1.5 mM of inorganic phosphate. Regular Advanced MEM medium contains 1 mM phosphate. As indicated, 2.5–5 µg/ml of either ELDL or control BSA was added and cells were cultured for up to 6 days with 0.5% FBS added every day. Cells were fixed by air drying at room temperature, calcium deposits were visualized by staining with Alizarin Red S. Before use, the pH of Alizarin Red S solution (2% w/v in distilled water) was adjusted to 4.1–4.4 using 10% NH4 OH. Fixed cells were stained with Alizarin Red S solution for 10 minutes and washed 3 times with distilled water to remove unbound stain. Quantification of the calcium-alizarin complexes were done according to a method described by Prosdocimo *et al*.^63^. Briefly, the deposited Alizarin-Ca^2+^ complexes were extracted by the addition of 1 ml of 10% CPC in water. This extract was further diluted 1:10 before the optical density was read at 570 nm.

### Human coronary artery smooth muscle cell proliferation

HCASMC at 3000 cells/ml were seeded into a 96 well plate. At indicated time points (24, 48, 72 and 120 h), MTS (3-(4,5-dimethylthiazol-2-yl)-5-(3-carboxymethoxyphenyl)-2-(4-sulfophenyl)-2H-tetrazolium) was added according to the manufacture’s protocol (Biovision, non-radioactive cell proliferation assay) and cells were incubated for 45 min. The reduction of MTS by the cells into a formazan product was measured directly at 590 nm using an Elisa plate reader (Fluostar, BMG labtech). For BrdU incorporation, cells (3000 cells/ml) were seeded onto glass cover slips and grown for 24 hours, and cells were pulsed with 10 μM BrdU (Sigma Aldrich) for 3 hours followed by detection of BrdU incorporation using anti BrdU monoclonal antibody (BD Biosciences) according to the manufactures. Nuclei were stained with DAPI. All nuclei and BrdU positive nuclei were counted on ten high power fields by a blinded investigator using a fluorescence microscope.

### Statistical analysis

All experiments were performed in triplicates and repeated two times. With the exception for the microarray experiment, which was performed on HCASMC from only one donor, all other experiments utilized three different batches of HCASMC (from Life technologies) and each cell line was tested in triplicates. Results are presented as mean ± standard deviation. Statistical differences were analyzed using independent sample T-test and one-way analysis of variance were used for mean comparison between two or multiple groups, respectively. Two-tailed probability values of p less than 0.05 were considered statistically significant for each test to ensure an overall study significance level of P < 0.05.

## Electronic supplementary material

Supplementary information

## References

[1] Torzewski M (2004). Enzymatic modification of low-density lipoprotein in the arterial wall: a new role for plasmin and matrix metalloproteinases in atherogenesis. Arterioscler Thromb Vasc Biol.

[2] Li D, Mehta JL (2005). Oxidized LDL, a critical factor in atherogenesis. Cardiovasc Res.

[3] Libby P (2002). Inflammation in atherosclerosis. Nature.

[4] Glass CK, Witztum JL (2001). Atherosclerosis. the road ahead. Cell.

[5] Allahverdian S, Chehroudi AC, McManus BM, Abraham T, Francis GA (2014). Contribution of intimal smooth muscle cells to cholesterol accumulation and macrophage-like cells in human atherosclerosis. Circulation.

[6] Vengrenyuk Y (2015). Cholesterol loading reprograms the microRNA-143/145-myocardin axis to convert aortic smooth muscle cells to a dysfunctional macrophage-like phenotype. Arterioscler Thromb Vasc Biol.

[7] Chellan B, Reardon CA, Getz GS, Hofmann Bowman MA (2016). Enzymatically Modified Low-Density Lipoprotein Promotes Foam Cell Formation in Smooth Muscle Cells via Macropinocytosis and Enhances Receptor-Mediated Uptake of Oxidized Low-Density Lipoprotein. Arterioscler Thromb Vasc Biol.

[8] Klouche M, Rose-John S, Schmiedt W, Bhakdi S (2000). Enzymatically degraded, nonoxidized LDL induces human vascular smooth muscle cell activation, foam cell transformation, and proliferation. Circulation.

[9] Bhakdi S (1995). On the pathogenesis of atherosclerosis: enzymatic transformation of human low density lipoprotein to an atherogenic moiety. J Exp Med.

[10] Twardowski L (2015). Enzymatically Modified Low-Density Lipoprotein Is Present in All Stages of Aortic Valve Sclerosis: Implications for Pathogenesis of the Disease. J Am Heart Assoc.

[11] Torzewski M (1998). Immunohistochemical demonstration of enzymatically modified human LDL and its colocalization with the terminal complement complex in the early atherosclerotic lesion. Arterioscler Thromb Vasc Biol.

[12] Kapinsky M (2001). Enzymatically degraded LDL preferentially binds to CD14(high) CD16(+) monocytes and induces foam cell formation mediated only in part by the class B scavenger-receptor CD36. Arterioscler Thromb Vasc Biol.

[13] Han SR (2003). Enzymatically modified LDL induces cathepsin H in human monocytes: potential relevance in early atherogenesis. Arterioscler Thromb Vasc Biol.

[14] Torzewski M, Lackner KJ (2006). Initiation and progression of atherosclerosis–enzymatic or oxidative modification of low-density lipoprotein?. Clin Chem Lab Med.

[15] Hakala JK (2003). Lysosomal enzymes are released from cultured human macrophages, hydrolyze LDL *in vitro*, and are present extracellularly in human atherosclerotic lesions. Arterioscler Thromb Vasc Biol.

[16] Sakurada T, Orimo H, Okabe H, Noma A, Murakami M (1976). Purification and properties of cholesterol ester hydrolase from human aortic intima and media. Biochim Biophys Acta.

[17] Oörni K (2004). Cysteine protease cathepsin F is expressed in human atherosclerotic lesions, is secreted by cultured macrophages, and modifies low density lipoprotein particles *in vitro*. J Biol Chem.

[18] Kovanen PT (1997). Chymase-containing mast cells in human arterial intima: implications for atherosclerotic disease. Heart Vessels.

[19] Schwartz SM, deBlois D, O’Brien ER (1995). The intima. Soil for atherosclerosis and restenosis. Circ Res.

[20] Virmani R, Burke AP, Farb A, Kolodgie FD (2006). Pathology of the vulnerable plaque. J Am Coll Cardiol.

[21] Newby AC (2007). Metalloproteinases and vulnerable atherosclerotic plaques. Trends Cardiovasc Med.

[22] Gerthoffer WT (2007). Mechanisms of vascular smooth muscle cell migration. Circ Res.

[23] Louis SF, Zahradka P (2010). Vascular smooth muscle cell motility: From migration to invasion. Exp Clin Cardiol.

[24] Johnson JL (2014). Emerging regulators of vascular smooth muscle cell function in the development and progression of atherosclerosis. Cardiovasc Res.

[25] Liu J, Ren Y, Kang L, Zhang L (2014). Oxidized low-density lipoprotein increases the proliferation and migration of human coronary artery smooth muscle cells through the upregulation of osteopontin. Int J Mol Med.

[26] Taylor J, Butcher M, Zeadin M, Politano A, Shaughnessy SG (2011). Oxidized low-density lipoprotein promotes osteoblast differentiation in primary cultures of vascular smooth muscle cells by up-regulating Osterix expression in an Msx2-dependent manner. J Cell Biochem.

[27] Staiger H (2009). Muscle-derived angiopoietin-like protein 4 is induced by fatty acids via peroxisome proliferator-activated receptor (PPAR)-delta and is of metabolic relevance in humans. Diabetes.

[28] Yoon JC (2000). Peroxisome proliferator-activated receptor gamma target gene encoding a novel angiopoietin-related protein associated with adipose differentiation. Mol Cell Biol.

[29] Kersten S (2000). Characterization of the fasting-induced adipose factor FIAF, a novel peroxisome proliferator-activated receptor target gene. J Biol Chem.

[30] Georgiadi A (2010). Induction of cardiac Angptl4 by dietary fatty acids is mediated by peroxisome proliferator-activated receptor beta/delta and protects against fatty acid-induced oxidative stress. Circ Res.

[31] Grygiel-Górniak B (2014). Peroxisome proliferator-activated receptors and their ligands: nutritional and clinical implications–a review. Nutr J.

[32] Kohno M (1997). Effect of natriuretic peptide family on the oxidized LDL-induced migration of human coronary artery smooth muscle cells. Circ Res.

[33] Zhu P, Goh YY, Chin HF, Kersten S, Tan NS (2012). Angiopoietin-like 4: a decade of research. Biosci Rep.

[34] Tan MJ, Teo Z, Sng MK, Zhu P, Tan NS (2012). Emerging roles of angiopoietin-like 4 in human cancer. Mol Cancer Res.

[35] Hruska KA, Mathew S, Saab G (2005). Bone morphogenetic proteins in vascular calcification. Circ Res.

[36] Nakagawa Y (2010). Paracrine osteogenic signals via bone morphogenetic protein-2 accelerate the atherosclerotic intimal calcification *in vivo*. Arterioscler Thromb Vasc Biol.

[37] Schurgers LJ, Cranenburg EC, Vermeer C (2008). Matrix Gla-protein: the calcification inhibitor in need of vitamin K. Thromb Haemost.

[38] Proudfoot D, Shanahan CM (2006). Molecular mechanisms mediating vascular calcification: role of matrix Gla protein. Nephrology (Carlton).

[39] Giachelli CM (2009). The emerging role of phosphate in vascular calcification. Kidney Int.

[40] Choi HY (2009). ATP-binding cassette transporter A1 expression and apolipoprotein A-I binding are impaired in intima-type arterial smooth muscle cells. Circulation.

[41] Katsuda S, Boyd HC, Fligner C, Ross R, Gown AM (1992). Human atherosclerosis. III. Immunocytochemical analysis of the cell composition of lesions of young adults. Am J Pathol.

[42] Kockx MM (1998). Apoptosis and related proteins in different stages of human atherosclerotic plaques. Circulation.

[43] Smith EB (1990). Transport, interactions and retention of plasma proteins in the intima: the barrier function of the internal elastic lamina. Eur Heart J.

[44] Cheng F (2017). Selective p38α MAP kinase/MAPK14 inhibition in enzymatically modified LDL-stimulated human monocytes: implications for atherosclerosis. FASEB J.

[45] Rodríguez-Carballo E, Gámez B, Ventura F (2016). p38 MAPK Signaling in Osteoblast Differentiation. Front Cell Dev Biol.

[46] Hofmann Bowman MA, McNally EM (2012). Genetic pathways of vascular calcification. Trends Cardiovasc Med.

[47] Rutsch F (2003). Mutations in ENPP1 are associated with ‘idiopathic’ infantile arterial calcification. Nat Genet.

[48] Tintut Y, Patel J, Parhami F, Demer LL (2000). Tumor necrosis factor-alpha promotes *in vitro* calcification of vascular cells via the cAMP pathway. Circulation.

[49] Yutzey KE (2014). Calcific aortic valve disease: a consensus summary from the Alliance of Investigators on Calcific Aortic Valve Disease. Arterioscler Thromb Vasc Biol.

[50] Nsaibia MJ (2017). OxLDL-derived lysophosphatidic acid promotes the progression of aortic valve stenosis through a LPAR1-RhoA-NF-κB pathway. Cardiovasc Res.

[51] Köster A (2005). Transgenic angiopoietin-like (angptl)4 overexpression and targeted disruption of angptl4 and angptl3: regulation of triglyceride metabolism. Endocrinology.

[52] Goh YY (2010). Angiopoietin-like 4 interacts with integrins beta1 and beta5 to modulate keratinocyte migration. Am J Pathol.

[53] Huang Z (2016). The downregulation of ANGPTL4 inhibits the migration and proliferation of tongue squamous cell carcinoma. Arch Oral Biol.

[54] Georgiadi A (2013). Overexpression of angiopoietin-like protein 4 protects against atherosclerosis development. Arterioscler Thromb Vasc Biol.

[55] Aryal B (2016). ANGPTL4 deficiency in haematopoietic cells promotes monocyte expansion and atherosclerosis progression. Nat Commun.

[56] Stitziel NO (2016). Coding Variation in ANGPTL4, LPL, and SVEP1 and the Risk of Coronary Disease. N Engl J Med.

[57] Talmud PJ (2008). ANGPTL4 E40K and T266M: effects on plasma triglyceride and HDL levels, postprandial responses, and CHD risk. Arterioscler Thromb Vasc Biol.

[58] Cristofori P (2004). Reduced progression of atherosclerosis in apolipoprotein E-deficient mice treated with lacidipine is associated with a decreased susceptibility of low-density lipoprotein to oxidation. Int J Exp Pathol.

[59] Zanchetti A (2002). Calcium antagonist lacidipine slows down progression of asymptomatic carotid atherosclerosis: principal results of the European Lacidipine Study on Atherosclerosis (ELSA), a randomized, double-blind, long-term trial. Circulation.

[60] Ray JL, Leach R, Herbert JM, Benson M (2001). Isolation of vascular smooth muscle cells from a single murine aorta. Methods Cell Sci.

[61] Law RE (1996). Troglitazone inhibits vascular smooth muscle cell growth and intimal hyperplasia. J Clin Invest.

[62] Shioi A (1995). Beta-glycerophosphate accelerates calcification in cultured bovine vascular smooth muscle cells. Arterioscler Thromb Vasc Biol.

[63] Prosdocimo DA, Wyler SC, Romani AM, O’Neill WC, Dubyak GR (2010). Regulation of vascular smooth muscle cell calcification by extracellular pyrophosphate homeostasis: synergistic modulation by cyclic AMP and hyperphosphatemia. Am J Physiol Cell Physiol.

