# Editorial Expression of Concern: Enzyme-modified non-oxidized LDL (ELDL) induces human coronary artery smooth muscle cell transformation to a migratory and osteoblast-like phenotype

**DOI:** 10.1038/s41598-020-67308-8

**Published:** 2020-07-01

**Authors:** Bijoy Chellan, Elizabeth Rojas, Chunling Zhang, Marion A. Hofmann Bowman

**Affiliations:** 1 Department of Medicine, University of Chicago, Chicago, IL USA; 2 University of California, Los Angeles, CA USA; 3 Center for Research Informatics, University of Chicago, Chicago, IL USA; 4 Department of Medicine, University of Michigan, Ann Arbor, MI USA

Addendum to: *Scientific Reports* 10.1038/s41598-018-30073-w, published online 10 August 2018

The Editors are issuing an Editorial Expression of Concern for this article. Readers are alerted that concerns have been raised with respect to Fig. 6A where the panels presenting histology data for the ELDL10 and rANGPTL4 conditions are duplicated. The authors have informed us that the original data are not available. The histology data in Fig. 6A do not affect the conclusions of the article. All authors agree with this statement.

