# Supplementary material for: Enzyme-modified non-oxidized LDL (ELDL) induces human coronary artery smooth muscle cell transformation to a migratory and osteoblast-like phenotype
Source: Sci Rep. 2018 Aug 10;8:11954. doi: 10.1038/s41598-018-30073-w (PMC6086911; doi:10.1038/s41598-018-30073-w)
Supplement: Supplementary file 1 [file 41598_2018_30073_MOESM1_ESM.docx]

**Supplementary Figure 15**

Immunoblot showing characterization of human coronary artery smooth muscle cells (HCASMC) purchased from Life technologies and human aortic endothelial cells (HAEC) using antibodies for CD31 (for endothelial cell detection) and alpha smooth muscle actin (for SMC detection). Molecular weight (MW) as indicated

HAEC - Human aortic endothelial cells

HCASMC-Human coronary artery smooth muscle cells

**CD31 (MW: 135 kDa)**

**HAEC	HCASMC**

**β actin (MW: 40 kDa)**

**α SMA (MW: 45 kDa)**

**HAEC	HCASMC**

**β actin (MW: 40 kDa)**

Additional supplementary data for raw images:

**Immunoblot A:** Whole cell lysis extracts were immunoblotted and incubated with 1µg/ml IgG beta actin (RD system) and membrane was developed for 5 seconds. Molecular weight for beta actin is 40 kDa. Lane 1 (HCASMC treated with ELDL, 1µg whole cell lysate protein), lane 2 (HCASMC treated with BSA, 1µg whole cell lysate), lane 3 (HAEC, 10 µg whole cell lysate), lane 4 (HCASMC 10µg whole cell lysate). Lane 5 and 6 (HCASMC 1 µg whole cell lysate).

HCASMC, human coronary artery smooth muscle cells; HAEC, human aortic endothelial cells.

**Blot A	Lanes: #1	#2	#3	#4	#5	#6**

**β actin (MW: 40 kDa)**

**Immunoblot B:** Whole cell lysis extracts (10µ g protein per lane) were immunoblotted and incubated with 1µg/ml IgG alpha SMA (abcam) and membrane was developed for 7 seconds. Molecular weight for alpha SMA is 45 kDa. Lane 1 (HACE), lane 2 (HCASMC).

**Blot B	Lane: #1	#2**

**Alpha SM actin (MW: 45 kDa)**

**Immunoblot C:** Whole cell lysis extracts (1 µg protein per lane) were immunoblotted and incubated with 1µg/ml IgG ANGPLT4 (abcam ab 115798) and membrane was developed for 5 seconds. In lane 4, whole cell lysis extract was spiked with 25ng recombinant ANGPLT4 as positive control. Molecular weight for ANGPLT4 is 50 kDa. Lane 1-3 (HCASMC treated with ELDL), lane 4 (rANPLT4 25 ng, positive control), lane 5 (HCASMC treated with ELDL), lane 6-8 (HCASMC treated with BSA).

**Blot C	Lane: #1	#2	#3	#4	#5	#6	#7	#8**

**ANGPL4 (MW: 50 kDa)**

**Immunoblot D:** Whole cell lysis extracts (10µ g protein per lane) were immunoblotted and incubated with 1µg/ml IgG beta actin (RD system) and membrane was developed for 5 seconds. Molecular weight for beta actin is 40 kDa. Lane 1 (Human arterial endothelial cells, HAEC), lane 2 (Human coronary artery smooth muscle cells, HCASM).

**Blot D	Lanes: #1	#2**

**Beta actin (MW: 40 kDa)**

**Immunoblot E**: Whole cell lysis extracts (10µ g protein per lane) were immunoblotted and incubated with 1µg/ml IgG CD31 (RD system) and membrane was developed for 5 seconds. Molecular weight for CD31 is 135 kDa. Lane 1 (Human arterial endothelial cells, HAEC), lane 2 (HCASM), lane 3-4: empty-no sample loaded, lane 5: (HAEC), lane 6: HCASM, lane 7-8: empty-no sample loaded.

**Blot E**

**Lane: #1	#2	#3	#4	#5	#6	#7	#8**

**CD31 (MW: 135 kDa)**
